# Membrane interactions and self‐association of components of the Ess/Type VII secretion system of *Staphylococcus aureus*

**DOI:** 10.1002/1873-3468.12065

**Published:** 2016-02-03

**Authors:** Franziska Jäger, Martin Zoltner, Holger Kneuper, William N. Hunter, Tracy Palmer

**Affiliations:** ^1^ Divisions of Molecular MicrobiologySchool of Life SciencesUniversity of Dundee, DundeeUK; ^2^ Biological Chemistry and Drug DiscoverySchool of Life SciencesUniversity of Dundee, DundeeUK

**Keywords:** membrane protein complex, protein secretion, *Staphylococcusaureus*, Type VII secretion

## Abstract

The Ess/Type VII protein secretion system, essential for virulence of pathogenic *Staphylococcus aureus*, is dependent upon the four core membrane proteins EssA, EssB, EssC and EsaA. Here, we use crosslinking and blue native PAGE analysis to show that the EssB, EssC and EsaA proteins individually form homomeric complexes. Surprisingly, these components appear unable to interact with each other, or with the EssA protein. We further show that two high molecular weight multimers of EssC detected in whole cells are not dependent upon the presence of EsxA, EsxB or any other Ess component for their assembly.

## Abbreviations

**ATC**, anhydrotetracycline

**DDM**, *n*dodecyl‐β‐D‐maltoside

**DSS**, disuccinimidyl suberate

**Figure 1:**
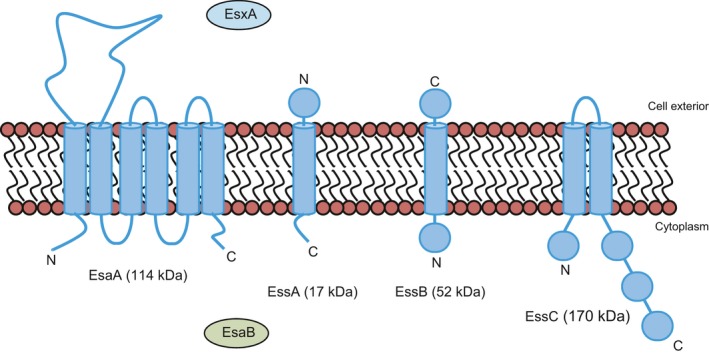
The membrane components of the *Staphylococcus aureus* Ess/Type VII protein secretion system. The sizes and predicted topologies of EsaA, EssA, EssB and EssC are shown. The other two essential components, EsxA and EsaB, are also shown, along with their known or predicted subcellular location.

Protein secretion systems allow bacteria to interact with and manipulate their environments, and in pathogens, they play critical roles in host colonisation and disease 1. The Gram‐positive bacterium *Staphylococcus aureus* produces a Type VII protein secretion system, also known as Ess (ESAT‐6 secretion system), that is required for pathogenesis in murine models of infection 2, 3. Substrates of the *S. aureus* Ess machinery include two small proteins, EsxA and EsxB, of the WXG100 superfamily, and two additional proteins, EsxC and EsxD, which lack the W‐X‐G motif 2, 4, 5. Mutational and bioinformatic analyses have revealed six core components of the *S. aureus* Ess machinery, of which four (EsaA, EssA, EssB and EssC) are predicted to be membrane‐bound proteins, one (EsaB) to be cytoplasmic and one (EsxA) extracellular [2, 3, 6; Fig 1]. Little is known about how the core Ess machinery is organised or how protein secretion is mediated.

The *S. aureus* Ess system is distantly related to the ESX/type VII secretion system of actinobacteria, sharing EsxA/B‐like and EssC‐like components. EssC proteins (termed EccC in actinobacteria) are membrane‐bound ATPases of the FtsK/SpoIIIE family that form hexameric membrane‐spanning pores which traffic macromolecules 7. Very recently, the structure of EccC from the thermophilic actinobacterium *Thermomonospora curvata* was reported. The protein has three interlocking ATPase domains at its C terminus and its multimerisation was unexpectedly shown to depend upon the binding of EsxB to a pocket on the most C‐terminal ATPase domain 8. In the same study, the structure of the two C‐terminal domains of EssC from *Geobacillus thermodenitrificans*, a thermophilic relative of *S. aureus* was also described, although it is not clear whether multimerisation of this protein is also controlled by interaction with EsxB (or EsxA) 8. Analysis of more than 150 *S. aureus* genome sequences has shown that the C‐terminal ATPase domain of EssC falls into one of four different sequence variants that cluster with genes coding cognate suites of candidate substrate proteins, implicating this domain in substrate recognition 6.

The EccC protein of pathogenic *Mycobacteria* has been shown to form a large (1.5 MDa) complex with three additional membrane proteins, EccB, EccD and EccE 9. However, these accessory proteins are found only in actinobacteria and it is not known whether *S. aureus* EssC forms part of a larger membrane‐bound complex containing additional components. To investigate this question, we probed the organisation of the essential membrane components of the *S. aureus* Ess system. Our results are consistent with three of the proteins, EsaA, EssB and EssC forming homo‐oligomeric complexes; however, we find no evidence for hetero‐oligomeric assemblies between any of these proteins or even with a fourth membrane‐bound protein, EssA, under the conditions we examined.

## Methods

### Plasmids and strains

*Staphylococcus aureus* strain RN6390 (WT; *ess* ^+^) 10 along with a series of isogenic strains lacking individual genes coded at the *ess* locus, or a deletion of all 12 of the coexpressed *ess* genes 3 were used for all experiments in this study. A plasmid construct producing N‐terminally hexahistidine‐tagged EsaA has been described previously 3. Plasmid pEssB‐nhis encodes N‐terminally hexahistidine‐tagged EssB was constructed as follows: the *essB* coding region was amplified from RN6390 genomic DNA using primers: 5′‐GATAGATCTGTTAAAAATCATAACCCTAAAAATG‐3′ and 5′‐CGAGAATTCACTATTTTTTT. CTTTCAGCTTCTTGGCGT‐3′, digested with *Bgl*II–*Eco*RI and cloned into the expression vector pRMC2h 3. A variant of plasmid pRMC2 11 coding for a C‐terminal hexahistidine tag (pRMC2ch) was generated by amplification across the multiple cloning site of pRMC2 using primers pRMC2seq1 (5′‐ATTTGGATCCCCTCGAGTTCATG‐3′) and Chisins (5′‐TTGAATTCATTAATGATGATGATGATGATGGAGCTCAGATCTGTTACC‐3′), digestion of the product with *Xho*I and *Eco*RI and cloning into similarly digested pRMC2. Plasmid pEssA‐chis encodes C‐terminally hexahistidine‐tagged EssA and was constructed following amplification of the *essA* coding region from RN6390 genomic DNA using primers: 5′‐CTAGATCTAATGTTACTTTTACGTGCTGATTCA‐3′ was digested with *Kpn*I and *Bgl*II and cloned into pRMC2ch. Plasmid pEssC‐chis encodes C‐terminally hexahistidine‐tagged EssC and was constructed by amplification of the *essC* coding region from RN6390 genomic DNA primers: 5′‐AAATAGATCTAGGACTGAGGCAAAG‐3 and 5′‐TGAATTCATTAATGATGATGATGATGAT. GACTACCAGATTTAAACCATCTAATCTTTTG‐3′ was digested with *Bgl*II and *Eco*RI and cloned into vector pRMC2.

### Biochemical methods

For *in vitro* crosslinking experiments with disuccinimidyl suberate (DSS), 50 mL of TSB medium was inoculated from an overnight culture of the strain of interest to give an OD_600_ of 0.1. For induction of expression of plasmid‐encoded proteins, the indicated concentration of anhydrotetracycline (ATC) was added when the OD_600_ of the culture reached 0.5, and the culture was incubated at 37 °C with vigorous shaking until an OD_600_ of 2.0 was reached. Cells were pelleted by centrifugation for 10 min at 2770 × ***g***, washed once with 1 mL 20 mm MOPS/NaOH, pH 7.2, 200 mm NaCl (M1 buffer), and resuspended in 1 mL M1 buffer containing 2.5 mm EDTA. The resuspended sample was supplemented with 200 μg lysostaphin and incubated for 30 min at 37 °C to digest the cell wall, after which sphaeroplasts were lysed by sonication and centrifuged for 5 min at 17 000 × ***g*** to remove unbroken cells. The clarified supernatant (containing the cytoplasm and membranes) was centrifuged again for 30 min at 227 000 × ***g*** at 4 °C to pellet the membranes. The membrane fraction was resuspended in 100 μL M1 buffer. For DSS crosslinking, membranes (30 μg of protein) were supplemented with either DSS (to a final concentration of 2 mm) or an equivalent volume of DMSO (control sample) and made up to 50 μL final volume with 20 mm HEPES/NaOH, pH 7.4, 20 mm KCl, 250 mm sucrose, 1 mm EDTA. After incubation for 30 min at room temperature, the reaction was quenched by addition of Tris/HCl, pH 8.0 to a final concentration of 100 mm. For analysis of EsaA or EssB crosslinks, samples were made up to 73 μL with 4 × NuPAGE loading buffer prior to separation on Bis‐Tris gels. For analysis of EssC crosslinks, samples were made up to 66 μL with 6 × SDS sample buffer prior to separation by SDS PAGE.

Formaldehyde (PFA) crosslinking experiments were carried out using whole cell samples. About 50 mL of TSB medium was inoculated from an overnight culture of the strain of interest to give an OD_600_ of 0.1. For induction of expression of plasmid‐encoded proteins, anhydrotetracycline (ATC; 50 ng·mL^−1^ for EsaA production, 100 ng·mL^−1^ for EssA production and 25 ng·mL^−1^ for EssB and EssC production) was added when the OD_600_ of the culture reached 0.5. Once an OD_600_ of 1.0 was reached, cells were pelleted by centrifugation for 10 min at 2770 × ***g*** and resuspended in 1 mL 1 × PBS. Paraformaldehyde was added to the cell suspension to a final concentration of 0.6% and samples incubated for 30 min at room temperature before the reaction was quenched by addition of Tris/HCl, pH 8.0 to a final concentration of 100 mm. Cells were pelleted by centrifugation for 10 min at 2770 × ***g***, resuspended in 1 mL 50 mm Tris/HCl, pH 7.5, 200 mm NaCl, 2.5 mm EDTA and treated as described above to obtain the isolated membrane fraction. Membranes were resuspended in 100 μL 50 mm Tris/HCl, pH 7.5, 200 mm NaCl prior to separation on Bis‐Tris gels (for analysis of EsaA or EssB crosslinks) or SDS gels (for analysis of EssC crosslinks).

Growth of cells and preparation of cell and supernatant samples for secretion experiments were performed as described previously 3. The same cell samples were also used to analyse the level of production of EsaA. To analyse the level of EssB and EssC, membrane fractions were prepared from whole cell samples as described above.

For detergent solubilisation tests, cells were harvested at OD_600_ of 2.0, following supplementation with ATC when the OD_600_ of the culture reached 0.5, conditions where the T7SS is active (Fig. S1). Membrane fractions were prepared as described above, and samples containing 400 μg of total protein were used for solubilisation. Membrane fractions were pelleted by centrifugation for 30 min at 227 000 × ***g*** at 4 °C and resuspended in solubilisation buffer (50 mm sodium phosphate, pH 8.0, 300 mm NaCl, 10% glycerol) to a final protein concentration of 10 μg·μL^−1^. Samples were supplemented with the appropriate detergent to a final concentration of 2%, and incubated at 25 °C for 2 h with shaking. To separate the solubilised protein from the insoluble material, samples were centrifuged for 20 min at 89 000 × ***g*** at 4 °C. The supernatant was taken as the solubilised membrane protein and the pellet as the insoluble material. For blue native polyacrylamide gel electrophoresis (BN PAGE), solubilised membrane protein samples were supplemented with 5% glycerol and 0.2% Coomassie Blue (final concentration) and BN PAGE was performed as described by Wittig *et al*. 12 using precast 4–16% gradient gels (Novex).

SDS PAGE was carried out using Bis‐Tris gels as described previously 3, and western blotting was performed following standard protocols with the following antibody dilutions: α‐EsxA 3 1 : 2500, α‐EsxC 3 1 : 2000, α‐EsaA 3 1 : 10 000, α‐EssB 3 1 : 10 000, α‐EssC 3 1 : 10 000 and α‐TrxA 13 1 : 10 000.

## Results

Currently little is known about the organisation of the membrane components of the *S. aureus* Ess machinery. A previous study has reported a membrane location for EssB in *S. aureus* 14 and when heterologously expressed in *E. coli* EssB behaves as a dimer 15, 16, but interactions with other components of the Ess machinery have not yet been described. We firstly used chemical crosslinking on isolated membrane fractions to probe the organisation of the essential membrane components EsaA, EssB and EssC, using the bifunctional crosslinker disuccinimidyl suberate (DSS), which crosslinks exposed primary amine‐containing residues. To maximise the possibility of detecting interactions, we also undertook these experiments with the overproduction or absence of individual Ess membrane components. Figure S1 confirms the lack of EsxA and EsxC secretion in each of the *esaA*,* essA*,* essB* and *essC* deletion strains and the complementation by the missing gene expressed *in trans* and Fig. S2 shows the level of plasmid‐encoded EsaA, EssB and EssC production.

**Figure 2:**
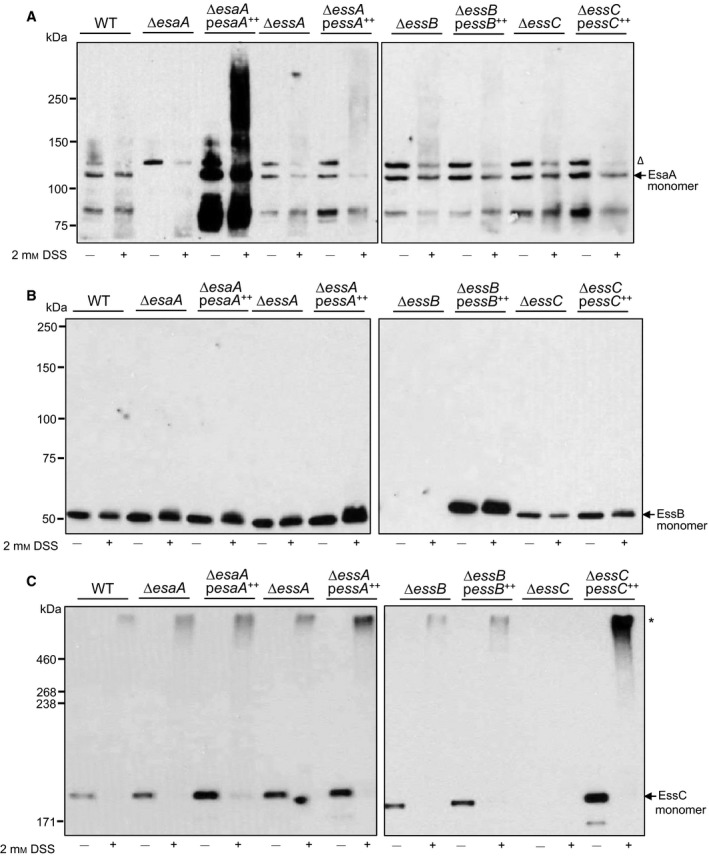
DSS‐mediated crosslinking of EsaA, EssB and EssC in isolated membrane fractions. Membrane fractions were prepared from strain RN6390 (WT) or the isogenic *esaA*,* essA*,* essB* or *essC* deletion strains, either harbouring the empty pRMC2 vector (lanes labelled ∆*esaA,* ∆*essA,* ∆*essB,* ∆*essC* respectively) or pRMC2 overproducing a his‐tagged variant of the indicated gene (∆*esaA pesaA* ^++^, ∆*essA pessA* ^++^, ∆*essB pessB* ^++^, ∆*essC pessC* ^++^) and treated with DSS as described under Experimental Procedures. After the reaction was quenched, aliquots (1 μg of total membrane protein for EsaA, EssB; or 2 μg of total membrane protein for EssC) were loaded on a Bis‐Tris gel containing 8% acrylamide (A and B) or SDS gel containing 5% acrylamide (C), samples were transferred to nitrocellulose membrane and proteins detected using polyclonal antibodies raised against A. EsaA, B. EssB or C. EssC, as indicated. Crosslinked products are indicated to the right with an asterisk; the open triangle indicates a nonspecific band that is detected with the EsaA antiserum.

As shown in Fig. 2(A), native EsaA could be detected in membranes derived from the wild‐type strain and each of the *essA*,* essB* and *essC* deletion strain backgrounds, indicating that it is stably produced in the absence of each of these Ess components. No additional EsaA‐specific bands were generated in the presence of the DSS crosslinker in membrane fractions from any of these strains, or when any of EssA, EssB or EssC was overproduced, although a dark smear of EsaA‐containing material was visible when crosslinking was carried out in the presence of overproduced EsaA. Figure 2(B) shows that likewise, EssB was stably produced and found in the membrane fraction in the absence of any of EsaA, EssA or EssC, but did not yield any crosslinked products, even when it was overproduced.

By contrast, crosslinked products were detected for EssC following incubation with DSS (Fig. 2C). A very similar high molecular weight band migrating well above the 460 kDa molecular weight marker was detected in membrane fractions of the wild‐type and the *esaA*,* essA* and *essB* deletion strains. It is likely that this represents a homo‐multimer of EssC, an observation consistent with previous reports 8. The mass of crosslinked product appears to be too large for a homo‐dimeric species (which would be expected to migrate below the 460 kDa marker) and therefore it is likely to represent at least a homo‐trimer. Note that we were unable to directly assess crosslinking of EssA as we do not have a functional antibody.

**Figure 3:**
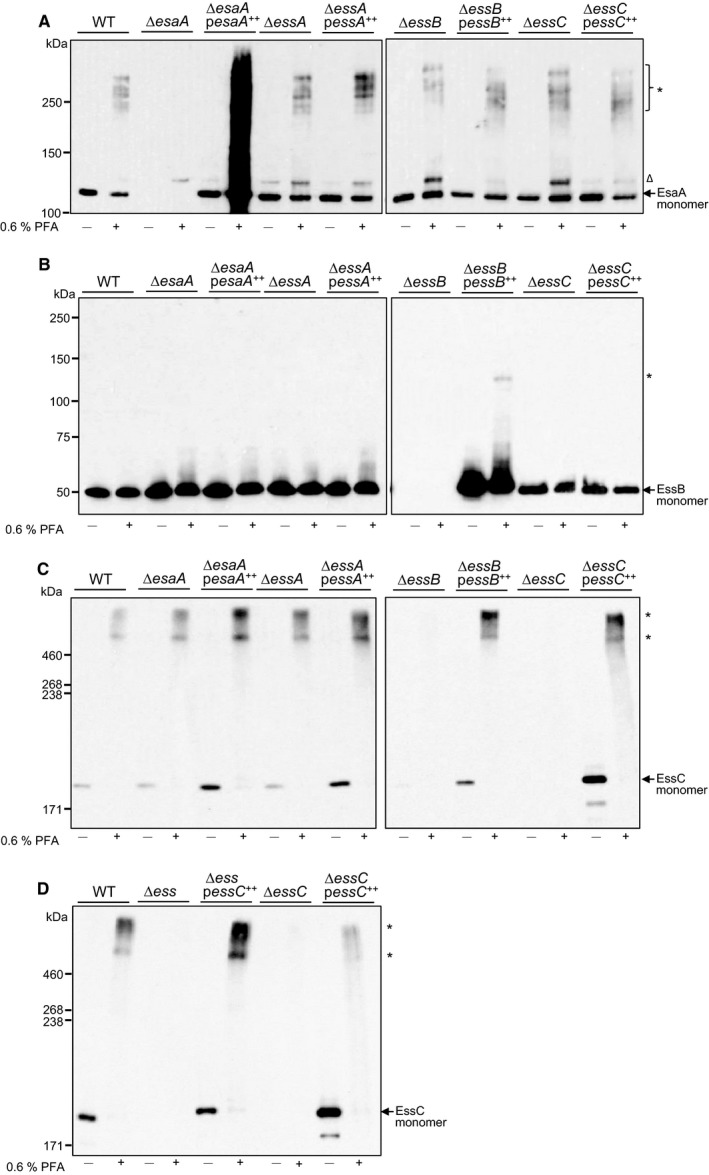
Formaldehyde‐mediated crosslinking of EsaA, EssB and EssC in whole cells. (A–C) Whole cells of strain RN6390 (WT) or the isogenic *esaA*,* essA*,* essB* or *essC* deletion strains, either harbouring the empty pRMC2 vector (lanes labelled ∆*esaA,* ∆*essA,* ∆*essB,* ∆*essC* respectively) or pRMC2 overproducing a his‐tagged variant of the indicated gene (∆*esaA pesaA* ^++^, ∆*essA pessA* ^++^, ∆*essB pessB* ^++^, ∆*essC pessC* ^++^) or (D) Whole cells of strain RN6390 (WT), the isogenic ∆*esaA* – *SAOUHSC_00269* deletion strain or the isogenic *essC* deletion strain, either harbouring the empty pRMC2 vector (lanes labelled ∆*ess* and ∆*essC* respectively) or pRMC2 overproducing a his‐tagged variant of EssC (∆*ess pessC* ^++^ or ∆*essC pessC* ^++^) were treated with paraformaldehyde (PFA) as described under Experimental Procedures. Following quenching, cells were lysed and membrane fractions prepared, and 1 μg of total membrane protein loaded on a Bis‐Tris gel containing 8% acrylamide (A and B) or 2 μg of total membrane protein loaded on a SDS gel containing 5% acrylamide (C and D), samples were transferred to nitrocellulose membrane and proteins detected using polyclonal antibodies raised against A. EsaA, B. EssB or C and D. EssC, as indicated. Crosslinked products are indicated to the right with an asterisk; the open triangle indicates a nonspecific band that is detected with the EsaA antiserum.

These initial crosslinking experiments, which revealed few detectable interactions, were carried out *in vitro*, where factors such as the proton‐motive force, ATP and soluble proteins are absent. Therefore, we next undertook similar crosslinking experiments in whole cells, using formaldehyde as a cell‐permeable crosslinker that also crosslinks amino groups. As shown in Fig. 3A, incubation with formaldehyde resulted in EsaA‐specific crosslinks in whole cells of the wild‐type strain. A series of 4–5 discrete bands could be detected, migrating around 250 kDa. Interestingly, essentially the same pattern of EsaA crosslinks was seen whether EssA, EssB or EssC were absent, suggesting that the crosslinked products did not contain any of these proteins. It has been reported that the long extracellular domain of YueB, the *Bacillus subtilis* homologue of EsaA, forms a highly elongated homo‐dimer 17 and it is therefore likely that the crosslinks seen here correspond to different conformers of a crosslinked EsaA dimer.

*In vivo* crosslinking analysis of EssB resulted in no detectable EssB crosslinks unless the protein was overproduced, in which case a very faint crosslinked product running at the approximate size of an EssB homo‐dimer could be detected (Fig 3B). As homo‐dimerisation of EssB has been reported previously, 15, 16 it is likely that this crosslink is a homo‐dimeric form of EssB.

Crosslinking of EssC *in vivo* also yielded high molecular weight products, as was observed *in vitro* (Fig 3C). However, in contrast to the *in vitro* analysis, where one major crosslinked product was seen, in this instance at least two distinct high molecular weight species were detected, both of which migrated above the 460 kDa molecular weight marker. The differences between the pattern of crosslinks seen *in vivo* and *in vitro* may reflect the difference in size between DSS which was used as a crosslinker *in vitro* and formaldehyde which was used *in vivo*. Alternatively they may arise due to the presence of ATP, proton‐motive force or other factors that are present in whole cells. It should be noted that as similar crosslinks were seen in the absence of any of EsaA, EssA or EssB, neither of the two crosslinks arise from interactions of EssC with any of the other core membrane components of the Ess system, and probably represent homo‐multimeric forms of EssC.

It has been reported that multimerisation of EccC, the homologue of EssC found in actinobacteria, is controlled by interaction with the two small WXG100 proteins, EsxA and EsxB. Rosenberg *et al*. 8 showed that the interaction of purified EccC with EsxB drives the formation of high‐order multimers, whereas interaction of EsxA with the EccC–EsxB complex resulted in cooperative disassembly of EccC and the accumulation of dimeric and monomeric species. To determine whether the high molecular weight EssC crosslinks were detected *in vivo* as a result of the presence of EsxA or EsxB, we repeated the formaldehyde crosslinking in whole cells of a strain deleted for all 12 genes encoded at the *ess* locus, including *esxA* and *esxB* 3. Figure 3D shows that the pattern of EssC crosslinks was not affected by the absence of EsxA, EsxB or any other protein encoded at this locus. We conclude that oligomerisation of EssC is independent of any previously identified Ess component.

**Figure 4:**
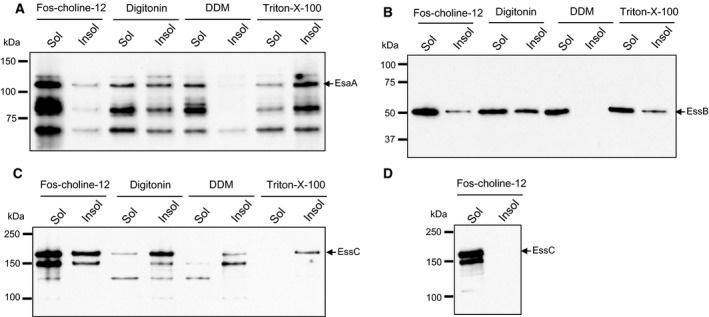
Detergent extraction of EsaA, EssB and EssC. Membrane fractions were treated with 2% of the indicated detergent, as described under Experimental Procedures. About 5 μg of solubilised (sol) and insoluble material (insol) following centrifugation for 20 min at 89 000 × ***g*** were loaded on a Bis‐Tris gel containing 8% acrylamide for (EsaA and EssC analysis) and 10% acrylamide (for EssB analysis). Subsequently the proteins were transferred on a nitrocellulose membrane followed by immunological detection of A. EsaA, B. EssB, C. and D. EssC.

The results presented to date suggest that there is likely homo‐oligomeric interactions for three of the membrane components of the Ess machinery, but provide no evidence for interactions between the components. One possible explanation for this is that there are no suitably juxtaposed lysine residues in neighbouring proteins to allow crosslinking to occur. Therefore, we next attempted to extract the Ess membrane proteins to examine interactions in detergent solution. We conducted solubilisation tests using the nonionic detergents Triton X‐100, *n*dodecyl‐β‐D‐maltoside (DDM) and digitonin, and the zwitterionic detergent Fos‐choline‐12, on membranes isolated from the *S. aureus* wild‐type strain. As shown in Figure 4, the EsaA, EssB and EssC proteins differed in their behaviour with the four detergents tested. DDM was able to successfully extract all of the EsaA and EssB from the membrane, but did not solubilise full length EssC. EsaA and EssB were also partially extracted with Triton X‐100 and digitonin, whereas only a very small fraction of EssC was extracted with digitonin and none when Triton X‐100 was used. The most effective detergent at extracting EssC was Fos‐choline‐12 which extracted between 50 and 100% of the EssC present in the membrane. This detergent was also able to extract most of the EssB and EsaA.

**Figure 5:**
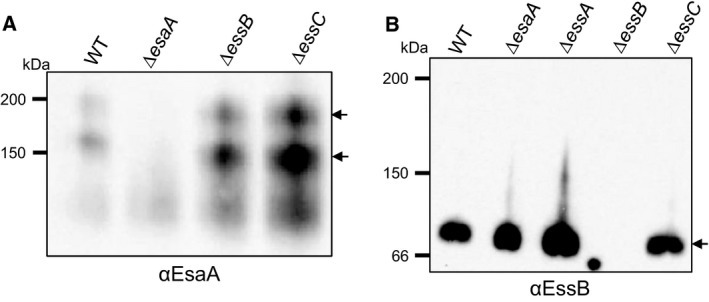
Blue native PAGE analysis of EsaA‐ and EssB‐containing complexes. Membrane fractions were prepared from strain RN6390 (WT) or the indicated deletion mutant, and samples (5 μg protein) were solubilised using 2% Fos‐choline‐12, as described under Experimental Procedures. The solubilised material was loaded onto a BN gel containing a gradient of 4–16% acrylamide. Subsequently, the proteins were transferred to PVDF membrane followed by immunological detection of A. EsaA or B. EssB. The arrows to the right indicate complexes containing EsaA (panel A) or EssB (panel B).

As Fos‐choline‐12 was able to extract all three proteins from the membrane, we next tested whether they were part of the same complex by undertaking blue native polyacrylamide gel electrophoresis (BN PAGE), a technique that is commonly used for the analysis of membrane protein complexes 12. After solubilisation of membranes with Fos‐choline‐12, the sample was separated on gels containing a gradient of 4–16% acrylamide and blotted with anti‐EsaA, anti‐EssB and anti‐EssC antibodies. Unfortunately, the anti‐EssC antibody was unable to detect any EssC‐specific bands following BN PAGE (data not shown). However, bands containing EsaA and EssB were observed. As shown in Fig. 5(A), two complexes containing EsaA could be detected, one of which close to the 150 kDa marker and the second just above the 200 kDa marker, which could conceivably correspond to monomers and dimers of EsaA. In contrast, a single EssB‐containing band was detected, migrating between the 66 kDa and 150 kDa marker, close to the expected size for an EssB dimer (Fig. 5B). Thus, given the differences in the masses of these bands these two proteins are highly unlikely to be part of the same complex. We confirmed this by repeating the BN PAGE on Fos‐choline‐12 membrane extracts from strains deleted for *esaA* or *essB*, and detected the same two EsaA complexes in the absence of EssB, and the same EssB complex in the absence of EsaA. We conclude that these two proteins do not interact with each other in isolated membranes.

Further analysis using membranes isolated from an *essC* strain background showed that the same two EsaA complexes could be detected in the absence of EssC (Fig. 5A). Likewise, an identically migrating EssB band was seen in membrane fractions derived from the *essC* and *essA* mutant strains (Fig. 5B). Taken together with the results of the crosslinking analysis and supported by prior observations of EssB behaviour by Zoltner *et al*. 15, 16, we conclude that the EsaA, EssB and EssC components of the Type VII secretion machinery show homotypic interaction but that there is no evidence that they are able to form complexes with one another, or with the fourth essential Ess membrane component, EssA, under the conditions we have examined.

## Discussion

In this study, we have probed the organisation of the *S. aureus* Ess machinery by examining complexes of the Ess membrane‐bound proteins using crosslinking and BN PAGE analysis. Our results are consistent with the EsaA, EssB and EssC proteins forming homomeric complexes, but under the conditions we examined, we found no evidence that these components interact with one another. Thus, we detected no heteromeric crosslinks between any of the proteins or with the fourth essential Ess membrane protein, EssA. Likewise the proteins displayed different solubility with a range of detergents, and only Fos‐choline‐12 of the detergents we tested was able to extract reasonable levels of EssC. BN PAGE analysis of membrane proteins extracted with Fos‐choline‐12 revealed EsaA and EssB to reside in separate complexes, neither of which appeared to contain any EssA or EssC. These findings contrast with a study of the distantly related ESX secretion system from *Mycobacterium marinum*, where extraction of membranes with DDM led to the isolation of a 1.5 MDa complex containing four conserved ESX membrane proteins, including the EssC homologue, EccC 9. However, there is no detectable homology between EccB, EccD and EccE, which along with EccC localise to the *M. marinum* ESX membrane complex, and EsaA, EssA or EssB, and it is possible that they may have unrelated functions in the two different secretion systems.

Structural and functional analysis of EccC has shown that multimerisation is driven by interaction with EsxB and conversely that EsxA promotes disassembly of the EccC multimer, most likely by binding to EsxB and forming an EccC–EsxB–EsxA ternary complex. A pocket within the most C‐terminal ATP‐binding domain of EccC binds the C‐terminal signal sequence of EsxB 8, providing structural clues about how multimerisation is controlled. However, it is not apparent whether this signal sequence binding pocket feature is conserved in EssC, and moreover we detected similar high‐order EssC multimers in a strain lacking EsxB and EsxA suggesting that oligomerisation is not dependent upon binding of any of the known *S. aureus* WXG100 proteins.

In conclusion, we see no evidence for the existence of a heteromeric Ess membrane complex. It remains possible that interactions between the membrane components are transient and depend upon the presence of a translocating substrate. An alternative explanation is that, under the conditions we have examined, the secretion machinery is not fully active, as it has been noted previously that Ess secretion is only poorly active under laboratory growth conditions 3. Further work will be required to distinguish between these possibilities.

## Author contributions

TP and WNH conceived and supervised the study; FJ designed and performed experiments; MZ and HK provided new tools and reagents; FJ, MZ, HK, WNH and TP analysed data; FJ and TP wrote the manuscript.

## Supporting information


**Fig. S1.** Complementation of *esaA*,* essA*,* essB* and *essC* deletion strains by provision of the missing gene *in trans*.

**Fig. S2**. Immunological detection of plasmid‐encoded EsaA‐his, EssB‐his and EssC‐his.
