# Supplementary material for: Membrane interactions and self‐association of components of the Ess/Type VII secretion system of Staphylococcus aureus
Source: FEBS Lett. 2016 Feb 3;590(3):349–57. doi: 10.1002/1873-3468.12065 (PMC4949537; doi:10.1002/1873-3468.12065)
Supplement: Supplementary file 1 — Fig. S1. Complementation of esaA, essA, essB and essC deletion strains by provision of the missing gene in trans. [file FEB2-590-349-s001.pptx]

## Slide 1
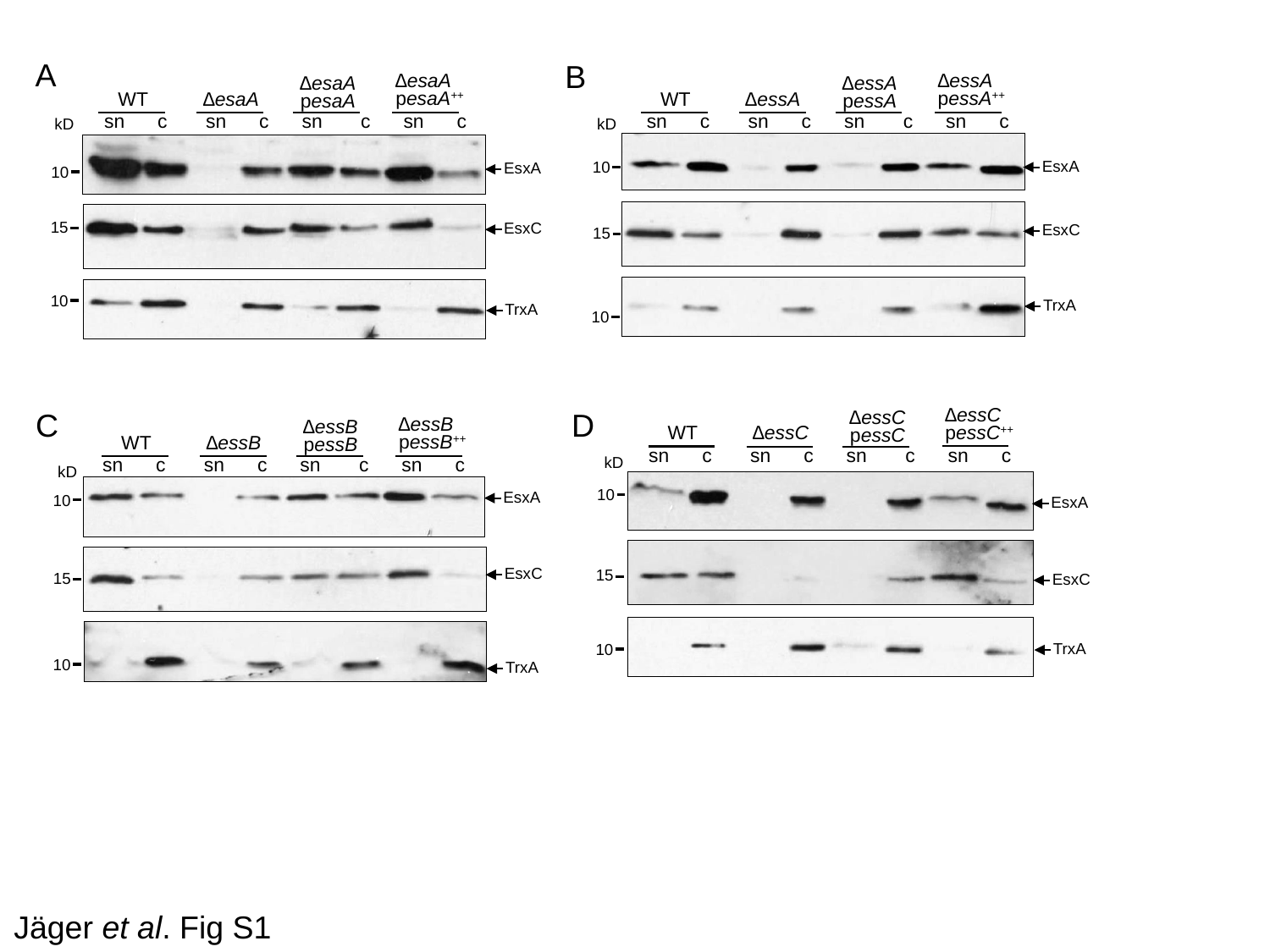

A
B
∆esaA
∆essA
∆esaA
∆essA
pesaA++
pessA++
∆esaA
∆essA
WT
WT
pesaA
pessA
sn c sn c sn c sn c
sn c sn c sn c sn c
kD
kD
EsxA
10
EsxA
10
15
EsxC
EsxC
15
10
TrxA
TrxA
10
∆essC
∆essC
pessC++
∆essC
WT
pessC
sn c sn c sn c sn c
kD
10
EsxA
15
EsxC
TrxA
10
D
C
∆essB
∆essB
pessB++
∆essB
WT
pessB
sn c sn c sn c sn c
kD
EsxA
10
EsxC
15
10
TrxA
Jäger et al. Fig S1
