# Supplementary material for: Membrane interactions and self‐association of components of the Ess/Type VII secretion system of Staphylococcus aureus
Source: FEBS Lett. 2016 Feb 3;590(3):349–57. doi: 10.1002/1873-3468.12065 (PMC4949537; doi:10.1002/1873-3468.12065)
Supplement: Supplementary file 2 — Fig. S2. Immunological detection of plasmid‐encoded EsaA‐his, EssB‐his and EssC‐his. [file FEB2-590-349-s002.pptx]

## Slide 1
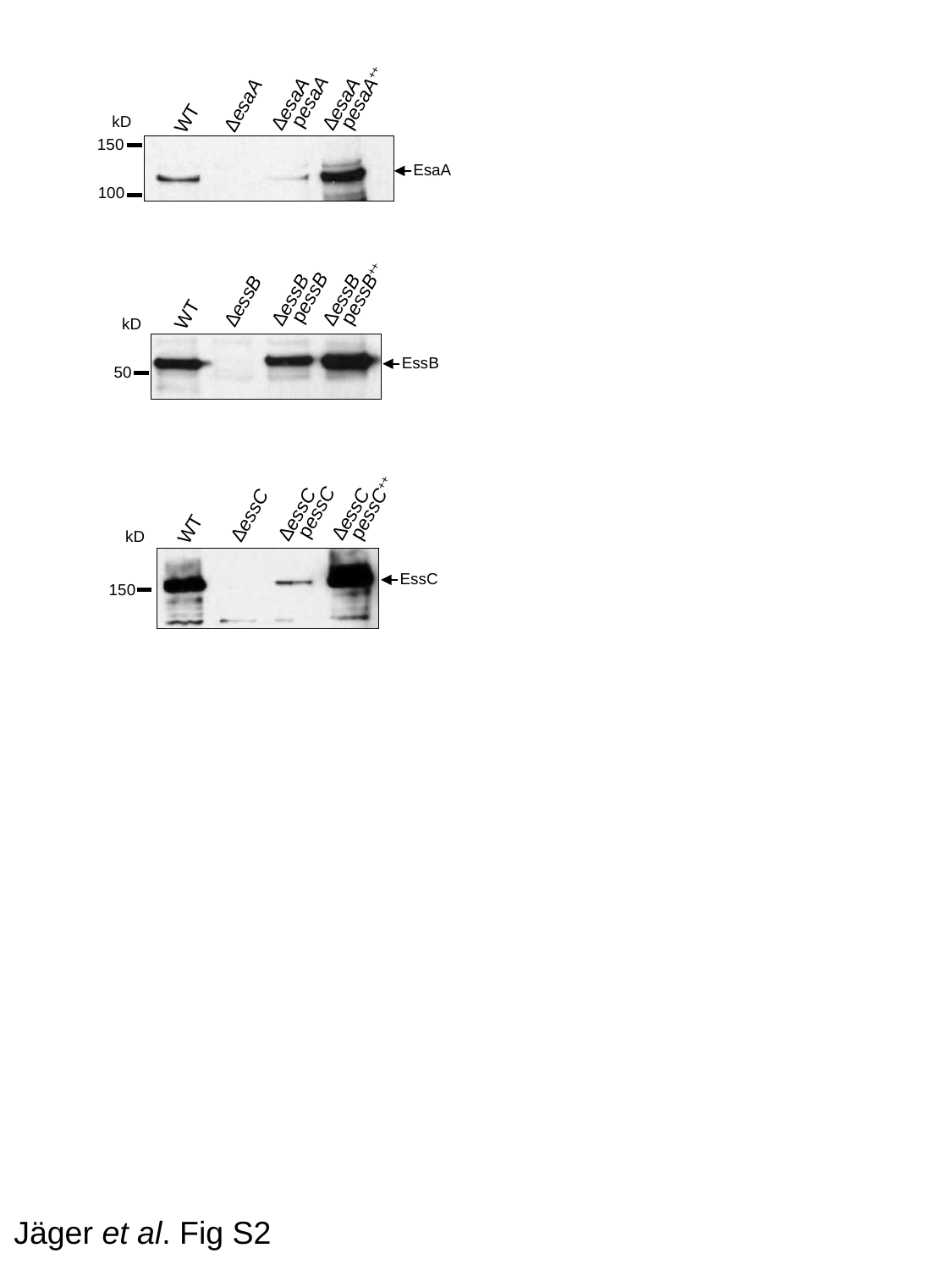

pesaA++
pesaA
∆esaA
∆esaA
∆esaA
WT
kD
150
EsaA
100
pessB++
pessB
∆essB
∆essB
∆essB
WT
kD
EssB
50
pessC++
pessC
∆essC
∆essC
∆essC
WT
kD
EssC
150
Jäger et al. Fig S2
