# Supplementary material for: Membrane interactions and self‐association of components of the Ess/Type VII secretion system of Staphylococcus aureus
Source: FEBS Lett. 2016 Feb 3;590(3):349–57. doi: 10.1002/1873-3468.12065 (PMC4949537; doi:10.1002/1873-3468.12065)
Supplement: Supplementary file 3 [file FEB2-590-349-s003.docx]

**Figure S1.** Complementation of *esaA*, *essA*, *essB* and *essC* deletion strains by provision of the missing gene *in trans*.

**Figure S2**. Immunological detection of plasmid-encoded EsaA-his, EssB-his and EssC-his.
